# Data from the batch adsorption of ciprofloxacin and lamivudine from synthetic solution using jamun seed (*Syzygium cumini*) biochar: Response surface methodology (RSM) optimization

**DOI:** 10.1016/j.dib.2023.108975

**Published:** 2023-02-13

**Authors:** Asha Ripanda, Mwemezi J. Rwiza, Elias Charles Nyanza, Ramadhani Bakari, Hossein Miraji, Karoli N. Njau, Said Ali Hamad Vuai, Revocatus L. Machunda

**Affiliations:** a School of Materials, Energy, Water and Environmental Sciences (MEWES), The Nelson Mandela African Institution of Science and Technology (NM-AIST), P.O. Box 447, Tengeru, Arusha, Tanzania; b Department of Chemistry, College of Natural and Mathematical Sciences, University of Dodoma, P.O. Box 338, Dodoma, Tanzania; c Department of Environmental and Occupational Health, School of Public Health, Catholic University of Health and Allied Sciences (CUHAS), Mwanza 1464, Tanzania; d Department of Petroleum and Energy Engineering, The University of Dodoma, P.O Box 11090, Dodoma, Tanzania

**Keywords:** Jamun seed (*Syzygium cumini*) biochar, Antimicrobial drug, Adsorption, Response surface, Environmental remediation, Biomass based adsorbents, Pollution

## Abstract

This dataset expresses the experimental data on the batch adsorption of ciprofloxacin and lamivudine from synthetic solution using jamun seed (JS) (*Syzygium cumini*) biochar. Independent variables including concentration of pollutants (10-500 ppm), contact time (30–300 min), adsorbent dosage (1-1000 mg), pH (1-14) and adsorbent calcination temperature (250,300, 600 and 750 °C) were studied and optimized using Response Surface Methodology (RSM). Empirical models were developed to predict the maximum removal efficiency of ciprofloxacin and lamivudine, and the results were compared with the experimental data. The removal of polutants was more influenced by concentration, followed by adsorbent dosagage, pH, and contact time and the maximum removal reached 90%.

**Specifications Table**

**Table:** 

Subject	Environmental sciences.
Specific subject area	Environmental chemistry.
Type of data	Figures, Tables, and surface plots.
How the data were acquired	Batch adsorption of ciprofloxacin and lamivudine on JS biochar, initial characterization by FTIR, BET and CHNS analysis. Analysis of residual pollutants from synthetic solution by UV-VIS.
Data format	Raw, Analyzed, Filtered.
Description of data collection	Jamun seeds were randomly collected from the environment, dried in the shade, ground, sieved and carbonized at the Nelson Mandela Institution of Science and Technology (NM-AIST), Tanzania. Initial characterization was done at The University of Dar es Salaam (UDOM), Tanzania, and adsorption experiments were done at The University of Dodoma, College of Natural and Mathematical sciences. The experiments were designed using RSM to build empirical models that could predict the removal efficiency of ciprofloxacin and lamivudine with high precision.
Data source location	N.A.
Data accessibility	Repository name: Mendeley data Data identification number: doi:10.17632/pphv3ygkfk.1. Direct URL to data: https://data.mendeley.com/datasets/pphv3ygkfk
Related research article	

## Value of the Data

• This dataset describes the potential of JS biochar for the removal of organic pollutants.
• This data can be used as a benchmark to compare the improvement of the adsorption of organics on JS biochar when the surface is activated using various additives.
• Researchers need to enhance ciprofloxacin and lamivudine removal further using analytical and soft computing tools.
• The process parameters, including pollutant concentration, adsorbent dose, contact time, pH, and calcination temperature, were optimized using the RSM tool. This approach significantly reduces the overall cost and time of doing experiments.
• The data in this study help in prediction of ciprofloxacin and lamivudine pollution removal as a result of excessive reagent use to investigate the removal of organic contaminants.

## 1 Objective

Antimicrobial drugs are linked with pollution and the development of resistant pathogens that may lead to treatment complications, rise hospitalization and death as well as threaten ecosystem and human health [1]. Reports indicate the presence of contaminants such as antibiotics in surface water, groundwater, effluents, and the entire ecosystem [1], [2], [3], [4], [5], [6], [7]. To ensure that the natural ecosystems are protected, it is necessary to generate data for policy reforms and search for potential adsorbents for removing contaminants such as antimicrobials from the environment. In this data set, the removal efficiency of ciprofloxacin and lamivudine from synthetic solution using JS biochar was investigated and reported.

## 2 Data Description

**Fig. 1:**
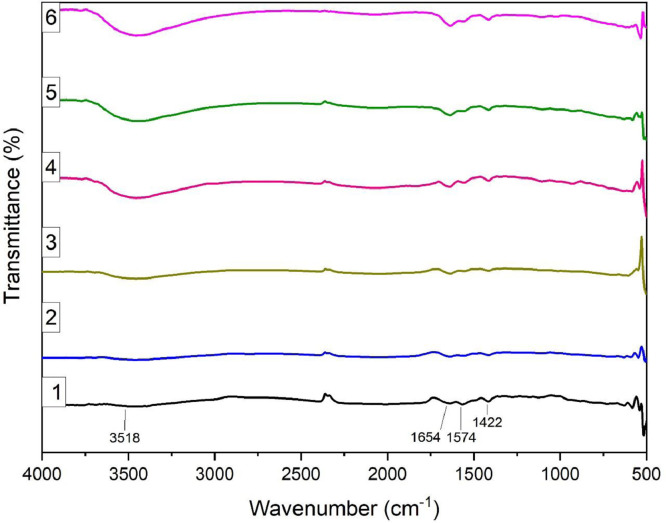
Functional groups present in JS biochar, Sample 1-6.

**Fig. 2:**
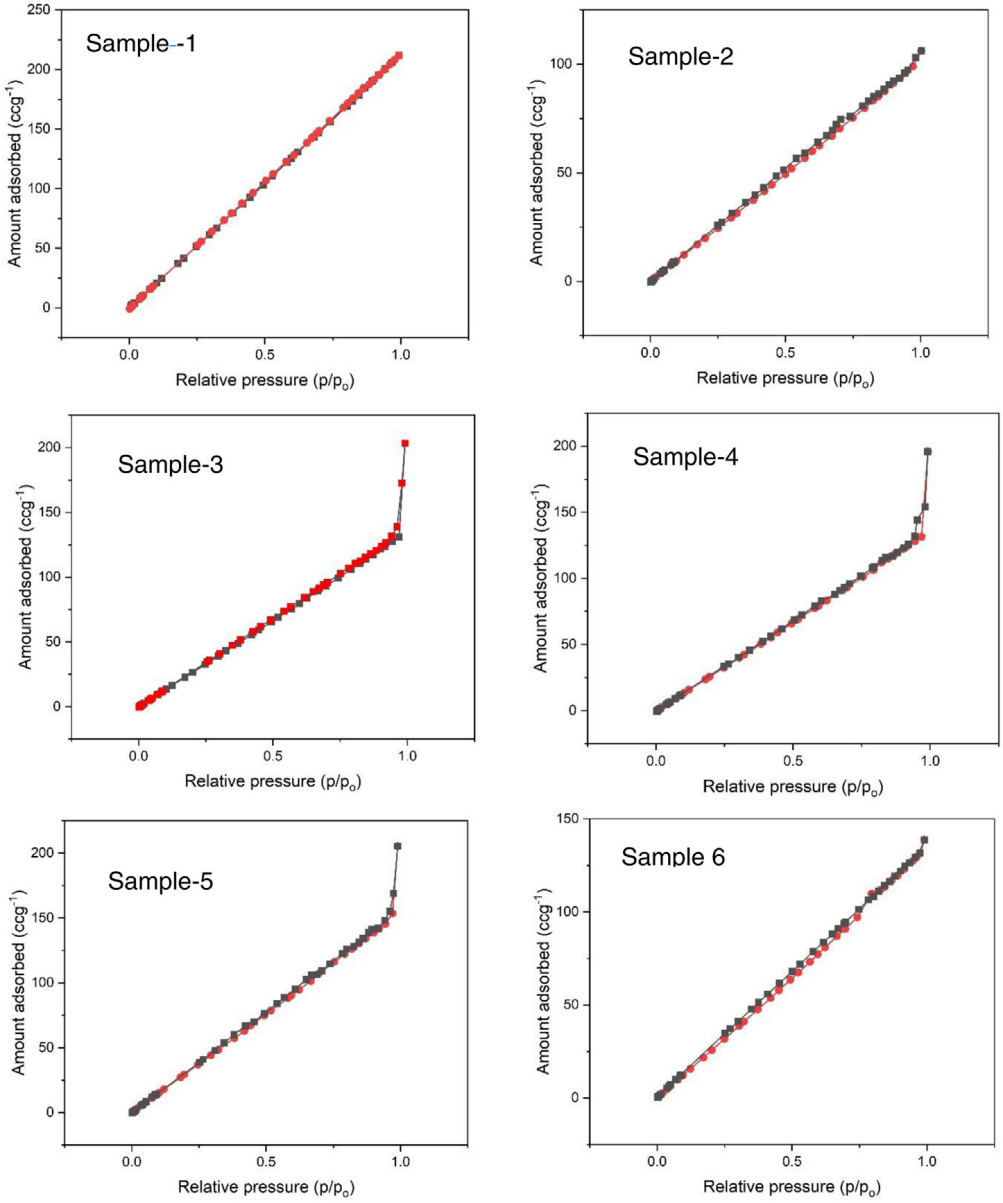
Presents adsorption isotherm of JS biochar Samples 1-6.

**Fig. 3:**
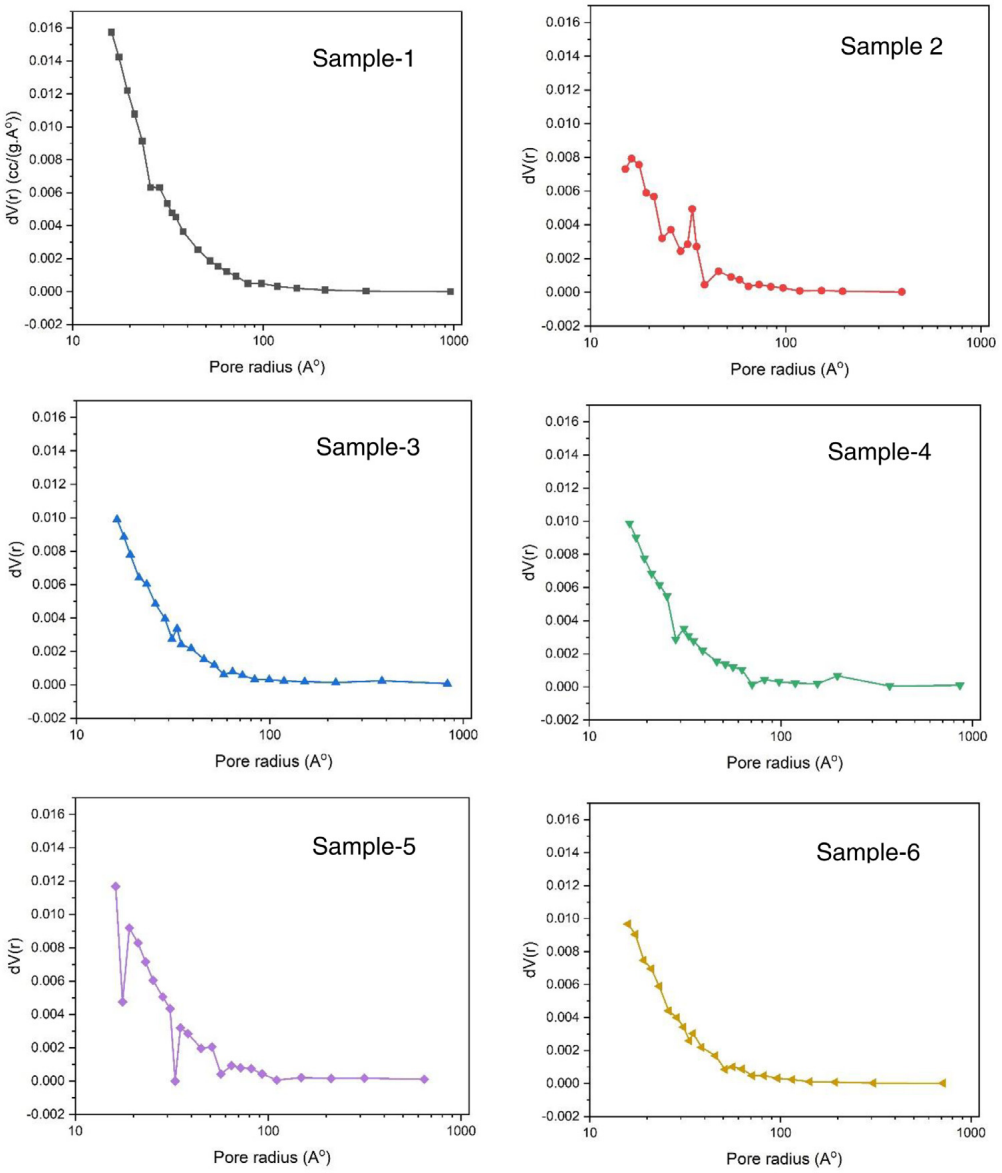
Pore size distribution of JS biochar.

Data sets generated are shared on Mendeley data [8]. The shared data on the removal of organics using JS biochar provides information on the preparation, initial characterization, experimental design and adsorption of ciprofloxacin and lamivudine from synthetic solution [8]. The results of the CHNS analysis are presented in Table 1. The results of FTIR (Fig. 1) show available functional groups that have potential interactions during the adsorption process. The broad band at around 3450 cm^−1^ to 3518 cm^−1,^ correspond to (OH^−^) hydroxyl groups. The peaks at 1422 cm^−1^, 1574 cm^−1^ and 1654 cm^−1^ may be due to C-H stretching (symmetrical for aliphatic and asymmetrical). The adsorption isotherms of the samples are presented in Fig. 2. Fig. 3 presents the pore size distribution of JS biochar material.

**Table 1:** Variation of carbon, nitrogen, and hydrogen in JS biochar samples.

Sample ID	Nitrogen %	Carbon %	Hydrogen %
1	1.69	58.39	4.56
2	1.87	64.24	3.86
3	2.01	77.25	3.43
4	2.08	79.38	2.71
5	2.29	87.93	2.54
6	1.4	76.61	1.07

**Fig. 4:**
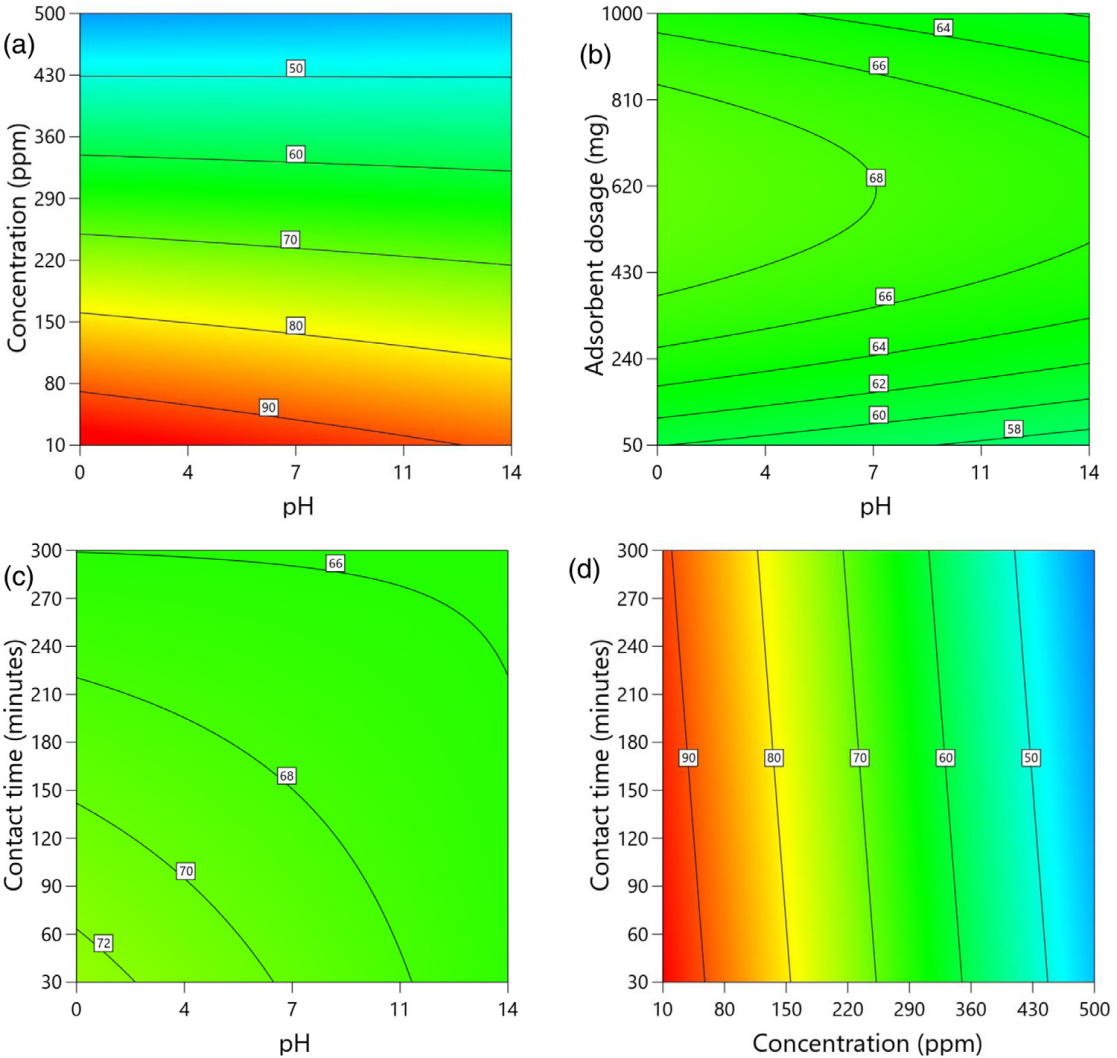
Variation of removal efficiency of ciprofloxacin by raw jamun seed biomass (a) effect of ciprofloxacin concentration and pH, (b) effect of adsorbent dosage and pH, (c)effect of contact time and pH, and (d) effect of contact time and ciprofloxacin concentration.

**Fig. 5:**
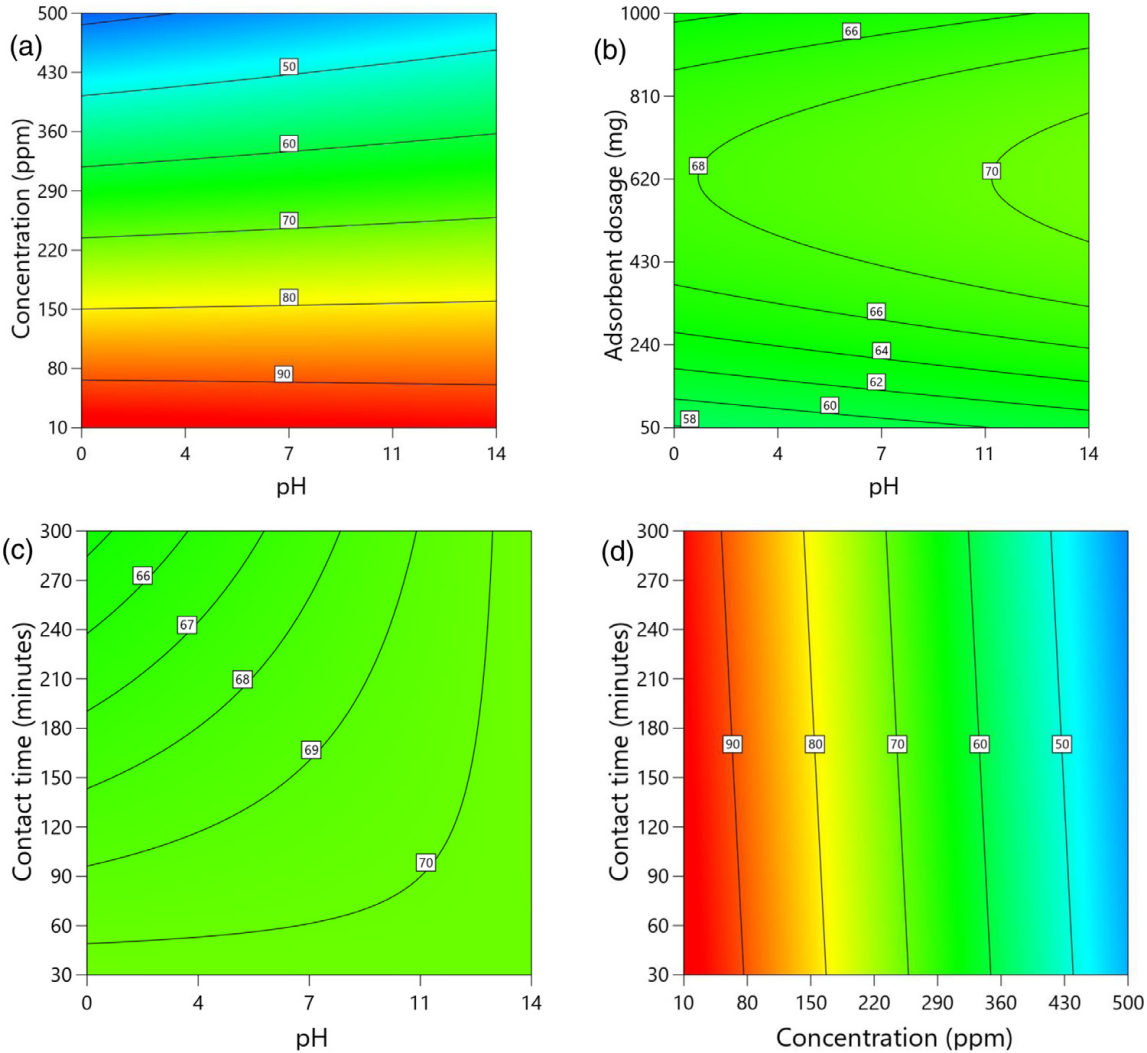
Variation of removal efficiency of ciprofloxacin by JS biochar calcined at 250°C (a) effect of ciprofloxacin concentration and pH, (b) effect of adsorbent dosage and pH, (c) effect of contact time and pH, and (d) effect of contact time and ciprofloxacin concentration.

**Fig. 6:**
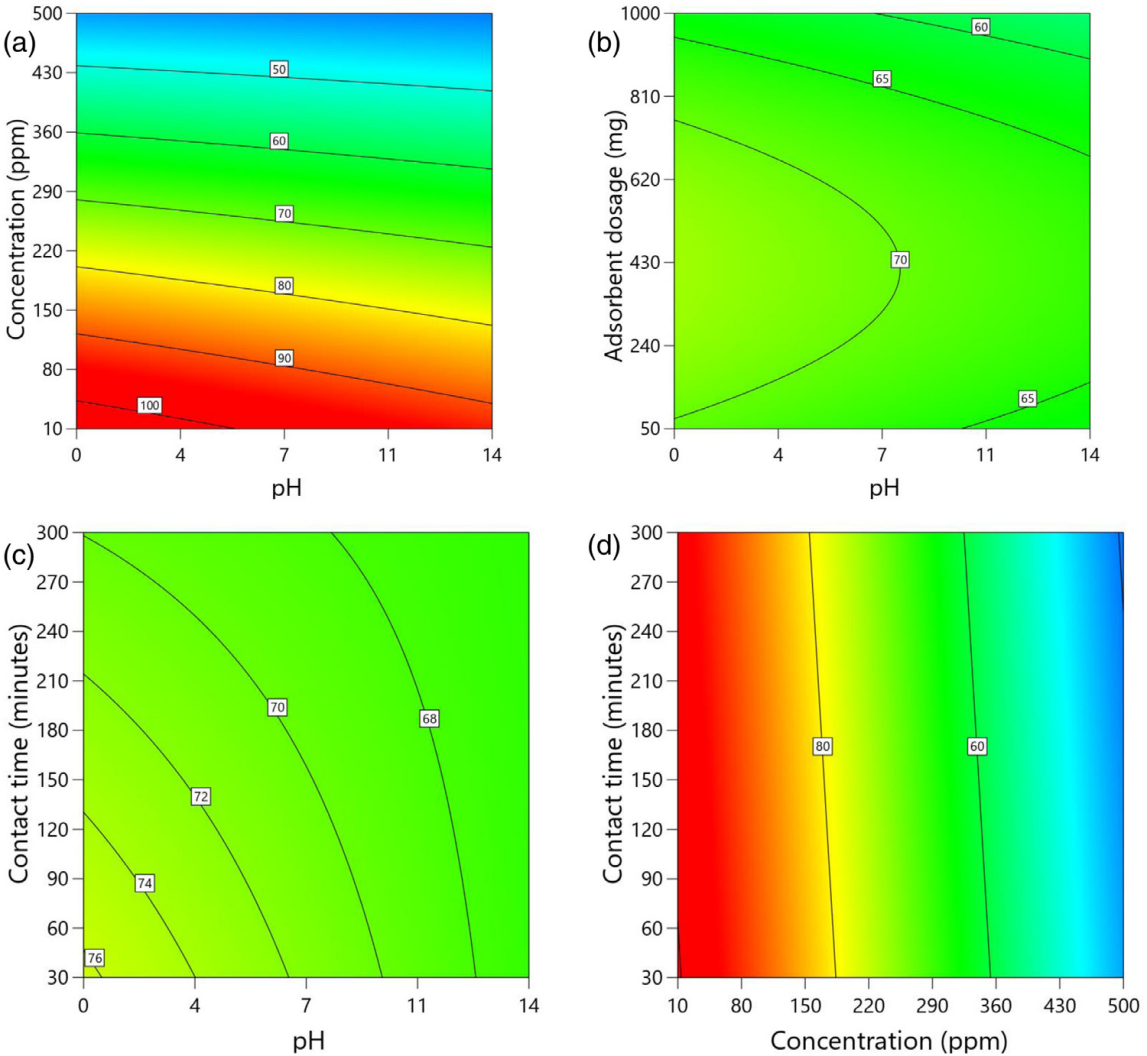
Variation of removal efficiency of ciprofloxacin by JS biochar calcined at 400°C (a) effect of ciprofloxacin concentration and pH, (b) effect of adsorbent dosage and pH, (c)effect of contact time and pH, and (d) effect of contact time and ciprofloxacin concentration.

**Fig. 7:**
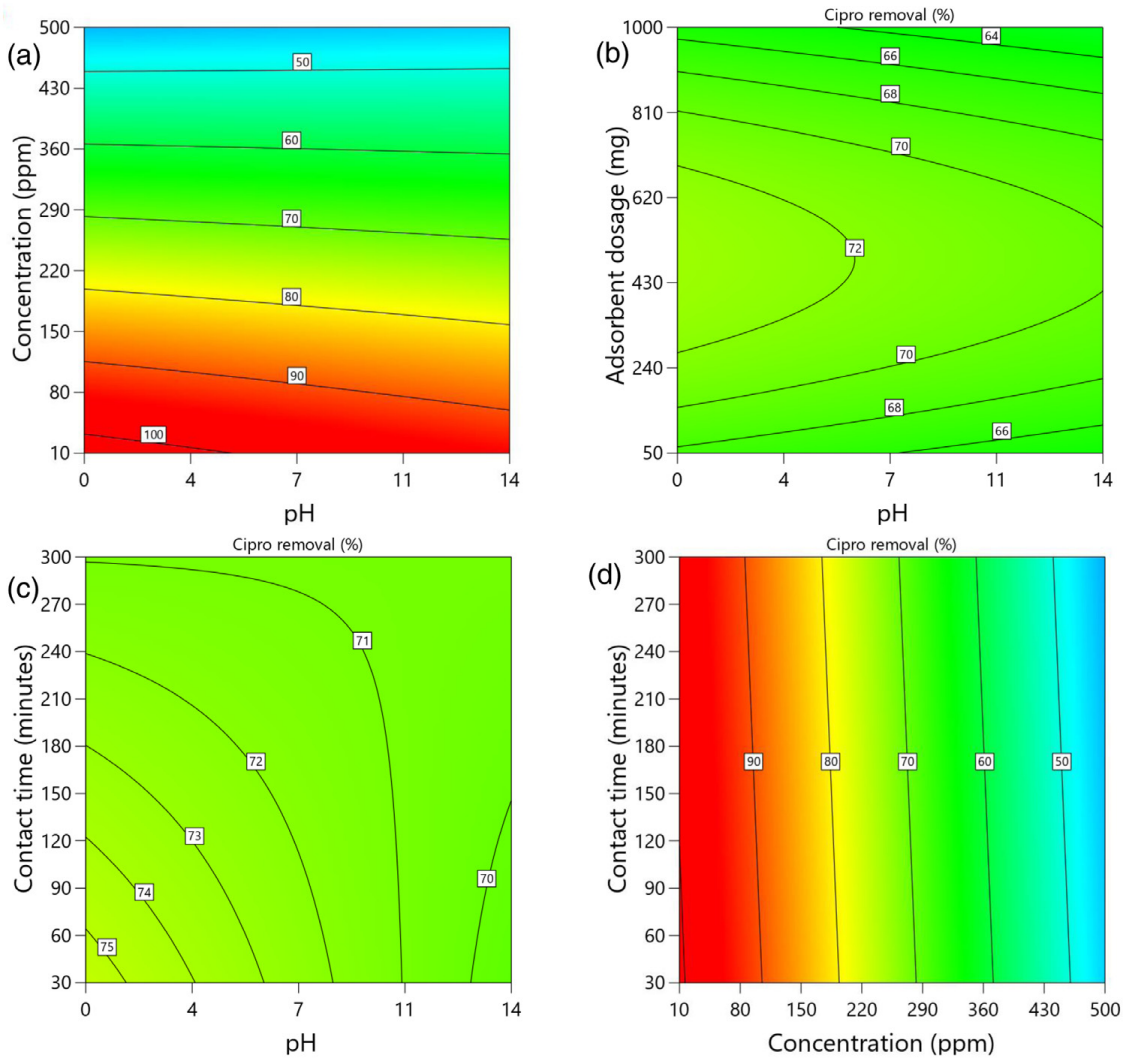
Variation of removal efficiency of ciprofloxacin by JS biochar calcined at 500°C (a) effect of ciprofloxacin concentration and pH, (b) effect of adsorbent dosage and pH, (c) effect of contact time and pH, and (d) effect of contact time and ciprofloxacin concentration.

**Fig. 8:**
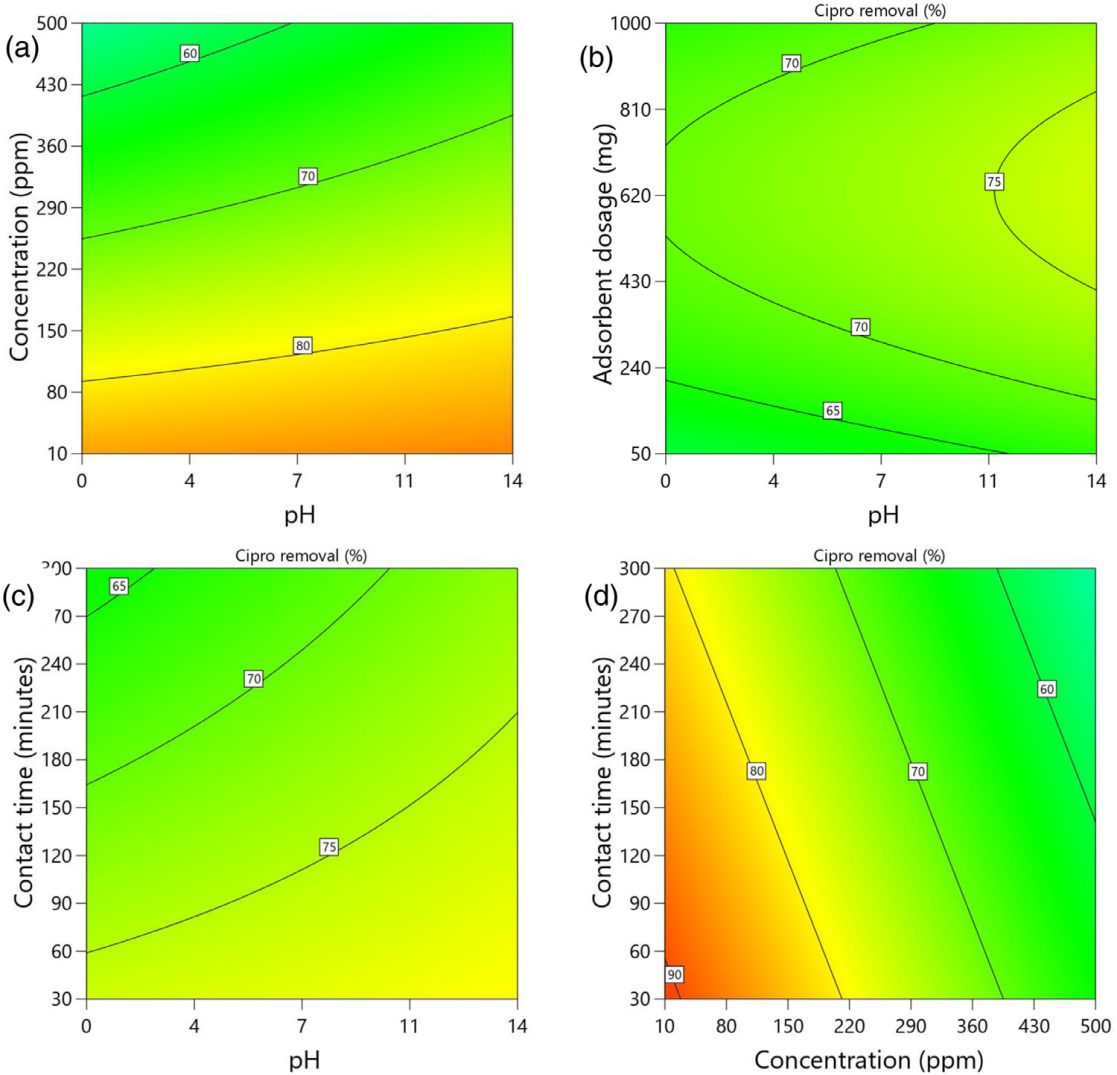
Variation of removal efficiency of ciprofloxacin by JS biochar calcined at 600°C (a) effect of ciprofloxacin concentration and pH, (b) effect of adsorbent dosage and pH, (c)effect of contact time and pH, and (d) effect of contact time and ciprofloxacin concentration.

**Fig. 9:**
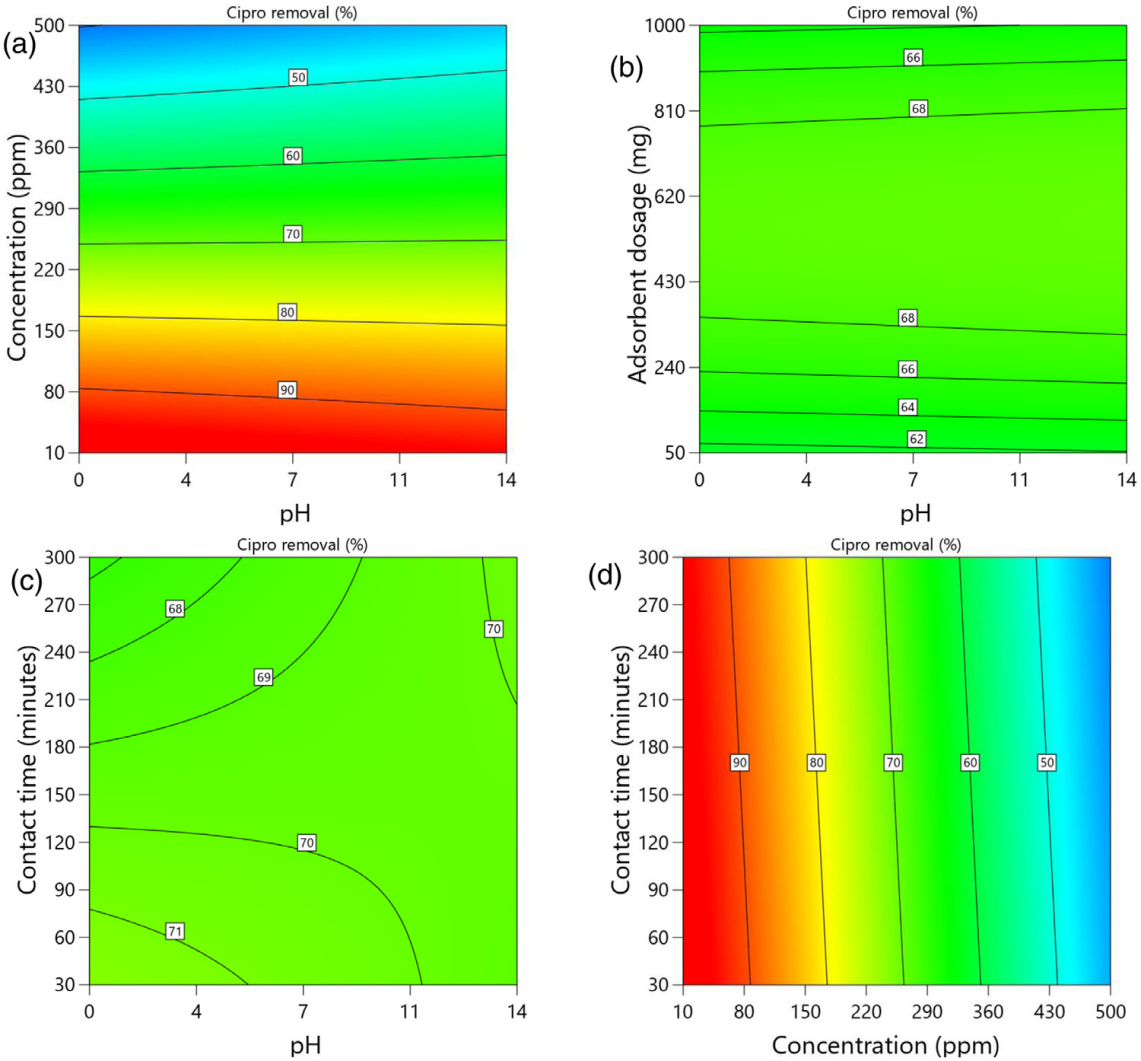
Variation of removal efficiency of ciprofloxacin by JS biochar calcined at 750°C (a) effect of ciprofloxacin concentration and pH, (b) effect of adsorbent dosage and pH, (c)effect of contact time and pH, and (d) effect of contact time and ciprofloxacin concentration.

**Fig. 10:**
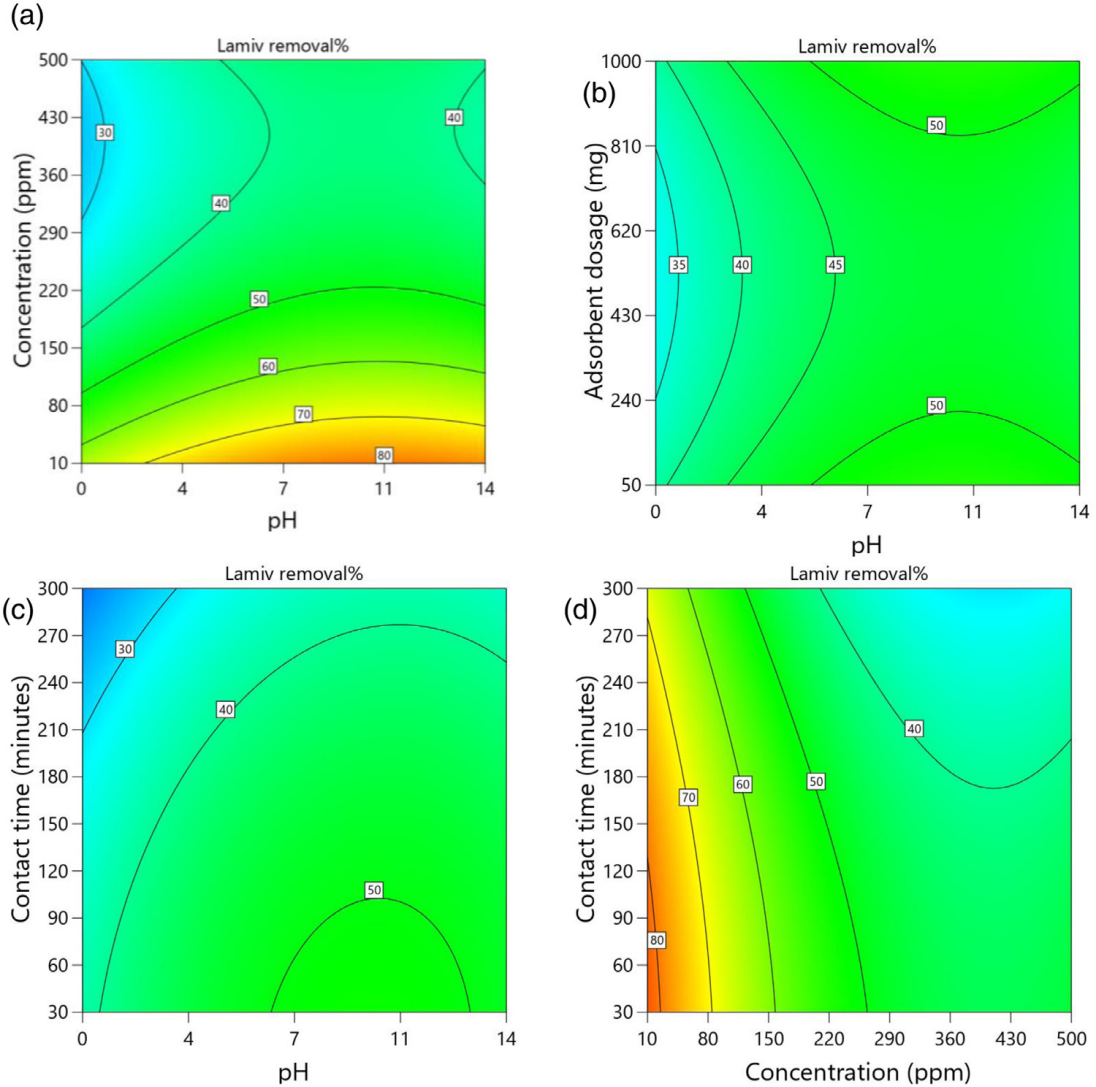
Variation of removal efficiency of lamivudine by raw JS biomass (a) effect of ciprofloxacin concentration and pH, (b) effect of adsorbent dosage and pH, (c) effect of contact time and pH, and (d) effect of contact time and ciprofloxacin concentration.

**Fig. 11:**
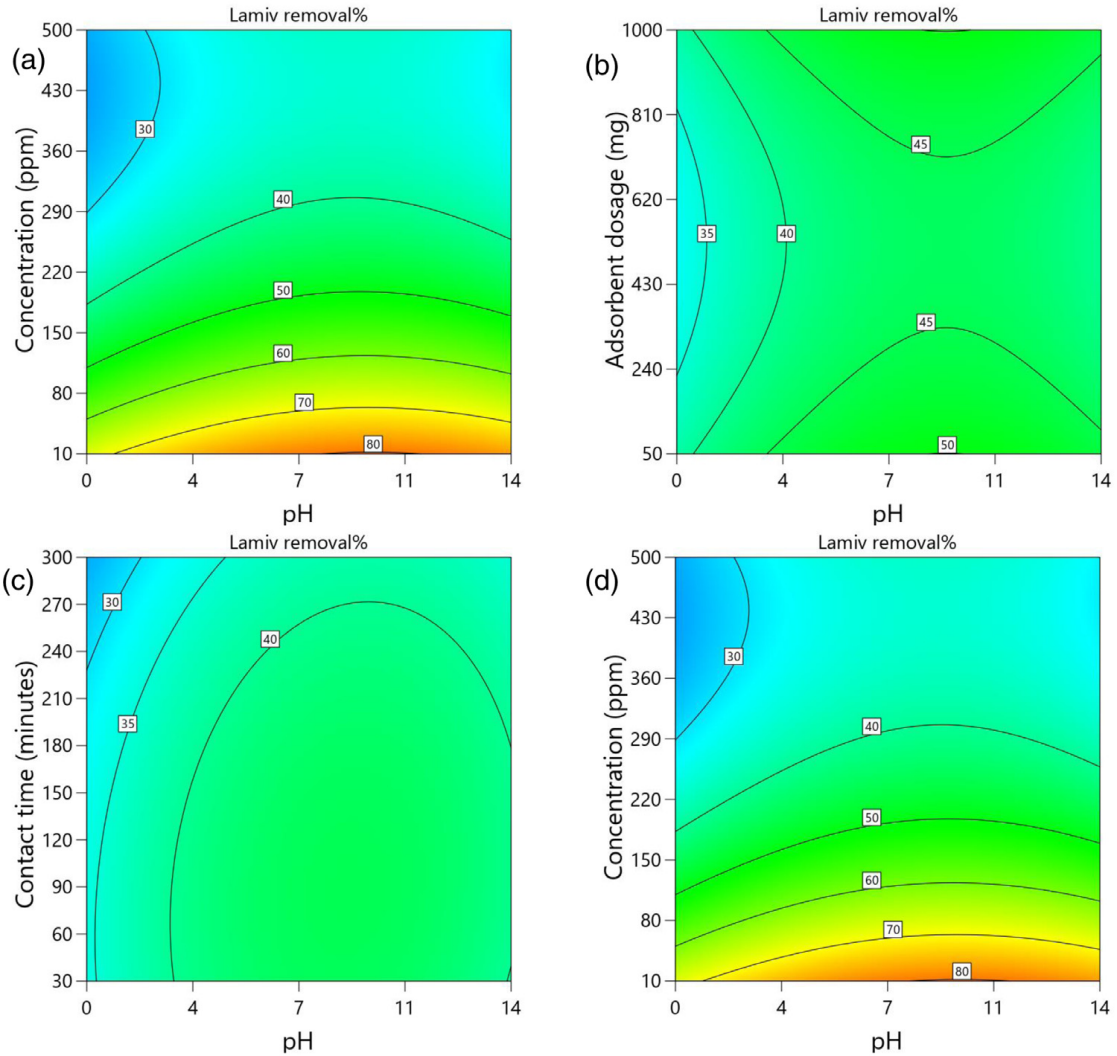
Variation of removal efficiency of lamivudine by JS biochar calcined at 250°C (a) effect of ciprofloxacin concentration and pH, (b) effect of adsorbent dosage and pH, (c) effect of contact time and pH, and (d) effect of contact time and ciprofloxacin concentration.

**Fig. 12:**
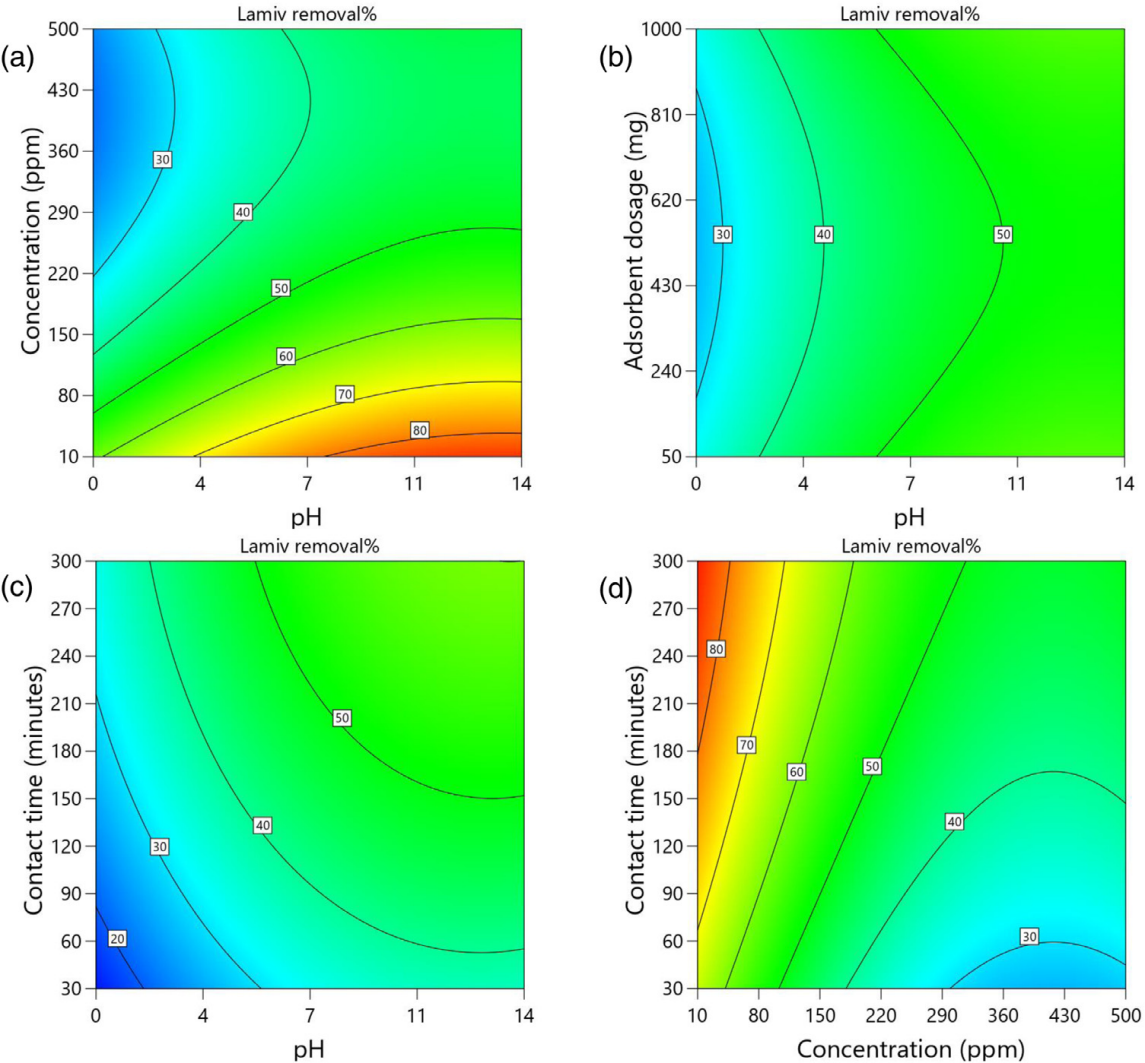
Variation of removal efficiency of lamivudine by JS biochar calcined at 400°C (a) effect of ciprofloxacin concentration and pH, (b) effect of adsorbent dosage and pH, (c) effect of contact time and pH, and (d) effect of contact time and ciprofloxacin concentration.

**Fig. 13:**
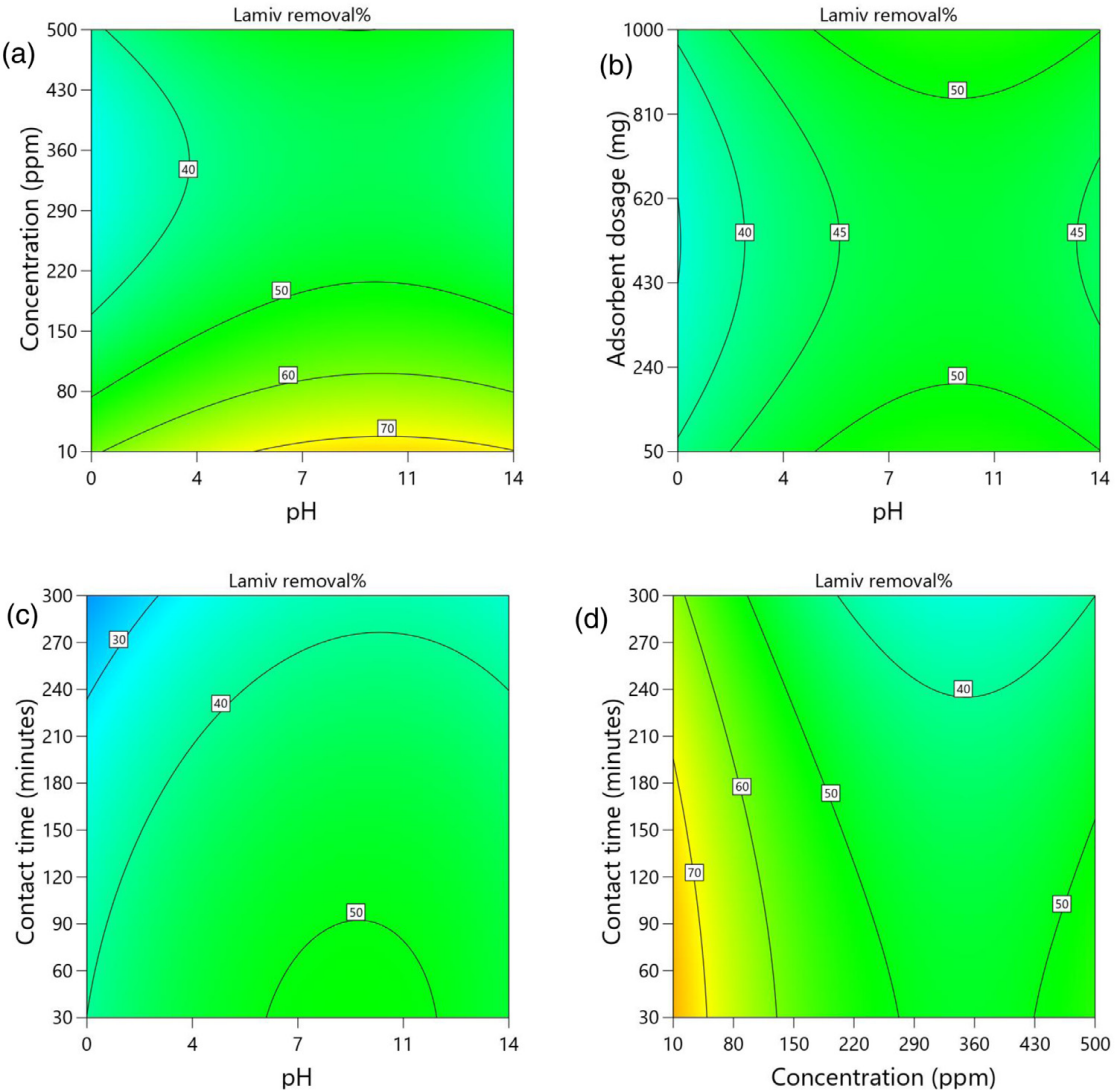
Variation of removal efficiency of lamivudine by JS biochar calcined at 500°C (a) effect of ciprofloxacin concentration and pH, (b) effect of adsorbent dosage and pH, (c)effect of contact time and pH, and (d) effect of contact time and ciprofloxacin concentration.

**Fig. 14:**
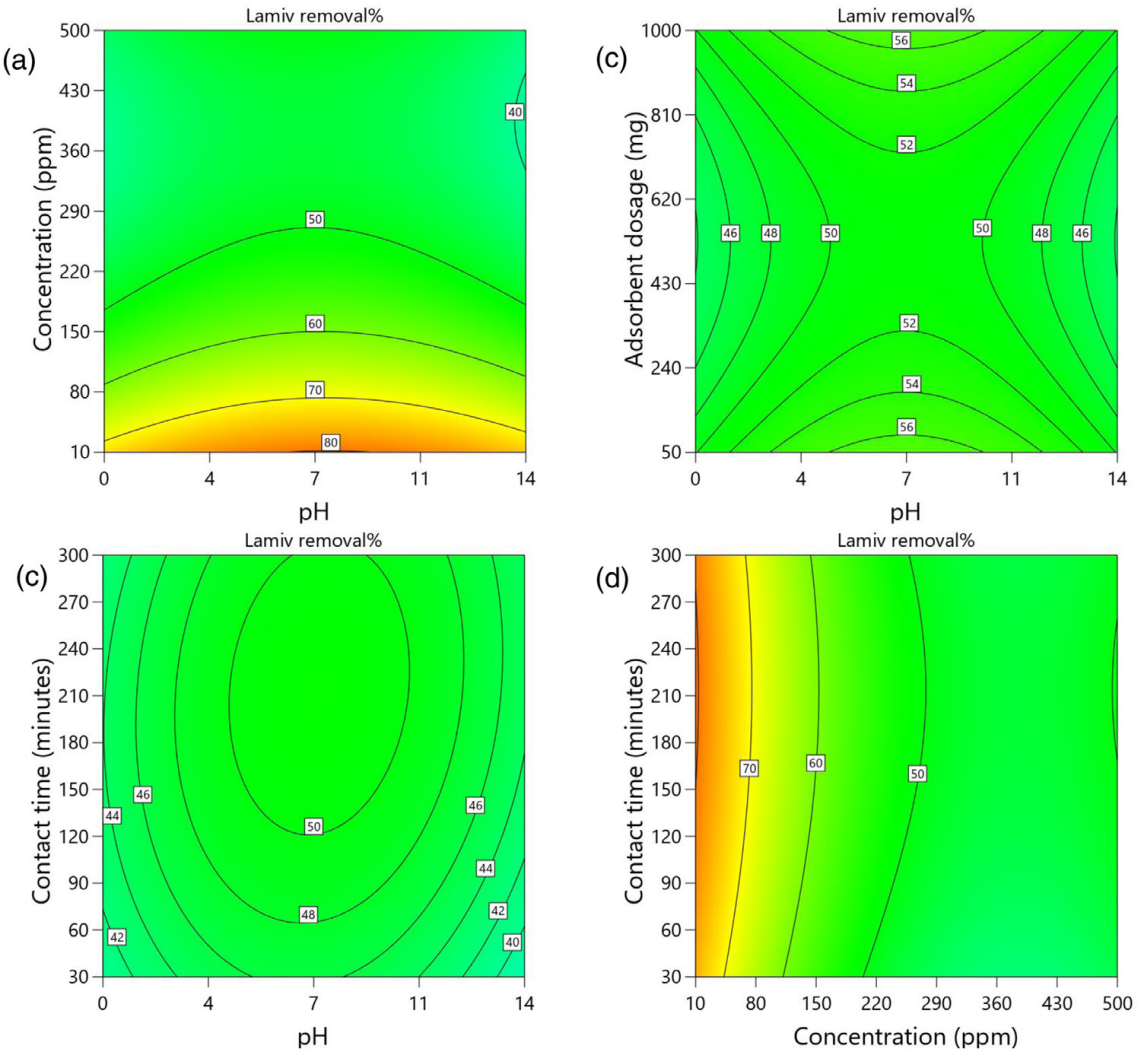
Variation of removal efficiency of lamivudine by JS biochar calcined at 600°C (a) effect of ciprofloxacin concentration and pH, (b) effect of adsorbent dosage and pH, (c) effect of contact time and pH, and (d) effect of contact time and ciprofloxacin concentration.

Tables 2 and 3 present the ANOVA results for a reduced quadratic model for the removal efficiency of ciprofloxacin and lamivudine, respectively. The R-squared of the model was close to one (R^2^ = 0.9968), implying that the data fitted well into the selected model. The predicted R² values were in reasonable agreement with the adjusted R² for both ciprofloxacin and lamivudine; the differences between predicted and adjusted R^2^ were less than 0. 2. Adequate precision measures the signal-to-noise ratio and a value greater than 4 is desirable. The ratio of 28.377 for ciprofloxacin and 36.910 for lamivudine indicated an adequate signal; therefore, this model can be used to navigate the design space. The suggested model gave a significant lack-of-fit (p-value less than 0.05), but other statistical parameters of the model were significant, and adequate precision is generally acceptable, thus allowing the model to be used for optimization purposes. Fig. 4, Fig. 5, Fig. 6, Fig. 7, Fig. 8, Fig. 9, Fig. 10, Fig. 11, Fig. 12, Fig. 13, Fig. 14 present the contour plots for the removal efficiency of ciprofloxacin and lamivudine from synthetic solution using JS biochar. The optimum removal efficiency of lamivudine (99.4%) was slightly higher compared to that of ciprofloxacin (99.1%) at different optimum conditions. These results indicate that the JS biochar may be used to remove organic contaminants from contaminated water and wastewater effluents.

**Table 2:** ANOVA values for a reduced quadratic model for ciprofloxacin.

Source	SS^a^	df	MS^b^	F-value	p-value	
Model	33911.95	38	892.42	130.43	< 0.0001	significant
A-pH	8.14	1	8.14	1.19	0.2916	
B-Concentration	22140.86	1	22140.86	3236.04	< 0.0001	
C-Adsorbent dosage	31.79	1	31.79	4.65	0.0467	
D-Contact time	35.16	1	35.16	5.14	0.0376	
E-Treatment temp	235.00	6	39.17	5.72	0.0024	
AB	97.83	1	97.83	14.30	0.0016	
AD	47.81	1	47.81	6.99	0.0177	
AE	174.94	6	29.16	4.26	0.0094	
BE	777.13	6	129.52	18.93	< 0.0001	
CD	165.44	1	165.44	24.18	0.0002	
CE	195.67	6	32.61	4.77	0.0057	
DE	128.82	6	21.47	3.14	0.0314	
C²	189.20	1	189.20	27.65	< 0.0001	
Residual	109.47	16	6.84			
Lack of Fit	108.89	11	9.90	85.17	< 0.0001	significant
Pure Error	0.5811	5	0.1162			
Cor Total	34021.42	54				

a SS is the Sum of Squares and ^b^MS is Mean Square.

**Table 3:** ANOVA values for a reduced quadratic model for lamivudine.

Source	SS^a^	df	MS^b^	F-value	p-value	
Model	17685.99	35	505.31	81.34	< 0.0001	significant
A-pH	553.75	1	553.75	89.13	< 0.0001	
B-Concentration	12335.31	1	12335.31	1985.56	< 0.0001	
C-Adsorbent dosage	1.43	1	1.43	0.2301	0.6369	
D-Contact time	14.02	1	14.02	2.26	0.1495	
E-Treatment temp	356.23	6	59.37	9.56	< 0.0001	
AB	23.42	1	23.42	3.77	0.0672	
AD	25.26	1	25.26	4.07	0.0581	
AE	872.00	6	145.33	23.39	< 0.0001	
BC	170.66	1	170.66	27.47	< 0.0001	
BE	368.46	6	61.41	9.88	< 0.0001	
DE	1033.85	6	172.31	27.74	< 0.0001	
A²	115.61	1	115.61	18.61	0.0004	
B²	703.90	1	703.90	113.30	< 0.0001	
C²	113.87	1	113.87	18.33	0.0004	
D²	25.95	1	25.95	4.18	0.0551	
Residual	118.04	19	6.21			
Lack of Fit	116.77	14	8.34	33.00	0.0006	significant
Pure Error	1.26	5	0.2527			
Cor Total	17804.02	54				

a SS is the Sum of Squares and ^b^MS is Mean Square.

### 2.1 Description of JS Biochar Samples

The samples were marked as Sample 1 (uncalcined, control), Sample 2 (calcined at 300 °C), Sample 3 (calcined at 400 °C), Sample 4 (calcined at 500 °C), Sample 5 (calcined at 600 °C), and Sample 6 (calcined at 750 °C).

### 2.2 Initial Characterization of JS Biochar

The percentage variation of carbon, nitrogen, and hydrogen in the prepared biochar are presented in Table 1.

The output of FTIR presenting available potential functional groups in JS biochar is presented in Fig. 1.

The adsorption isotherms of JS biochar samples (1-6) are presented in Fig. 2.

The pore size distribution of JS biochar Samples 1-6 is presented in Fig. 3.

Batch adsorption experiments using JS biochar were used to generate data on the removal efficiency of ciprofloxacin and lamivudine. Tables 2 and 3 present the ANOVA results for a reduced quadratic model for the removal efficiency of ciprofloxacin and lamivudine.

### 2.3 The Removal Efficiency of Ciprofloxacin

The removal efficiency of ciprofloxacin is presented in Fig. 4, Fig. 5, Fig. 6, Fig. 7, Fig. 8, Fig. 9.

### 2.4 Removal of Lamivudine

The removal efficiency of lamivudine is presented in Figs. 10–14..

**Fig. 15:**
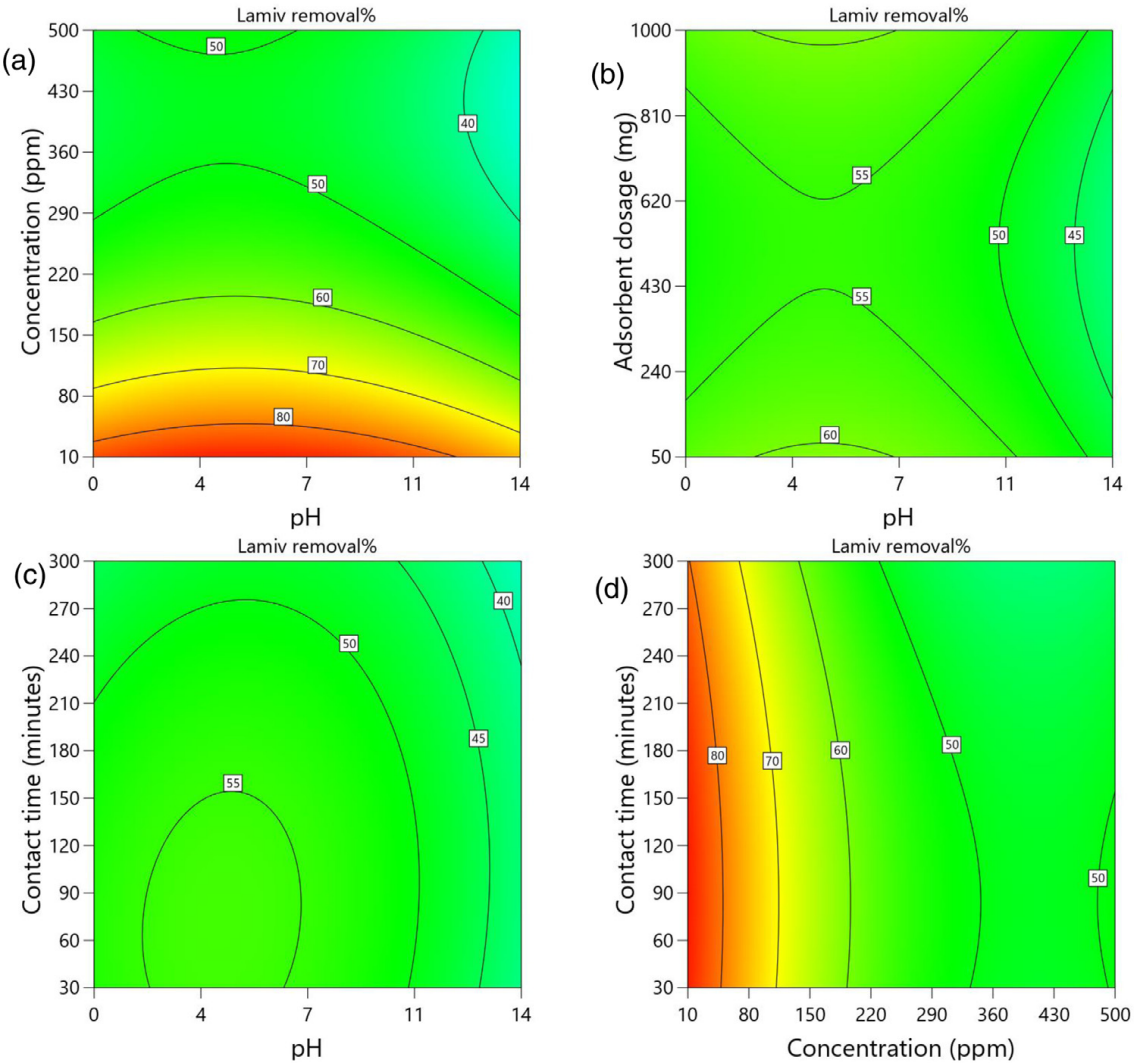
Variation of removal efficiency of lamivudine by JS biochar calcined at 750°C (a) effect of ciprofloxacin concentration and pH, (b) effect of adsorbent dosage and pH, (c) effect of contact time and pH, and (d) effect of contact time and ciprofloxacin concentration.

### 2.5 Optimization and Model Confirmation

**Fig. 16:**
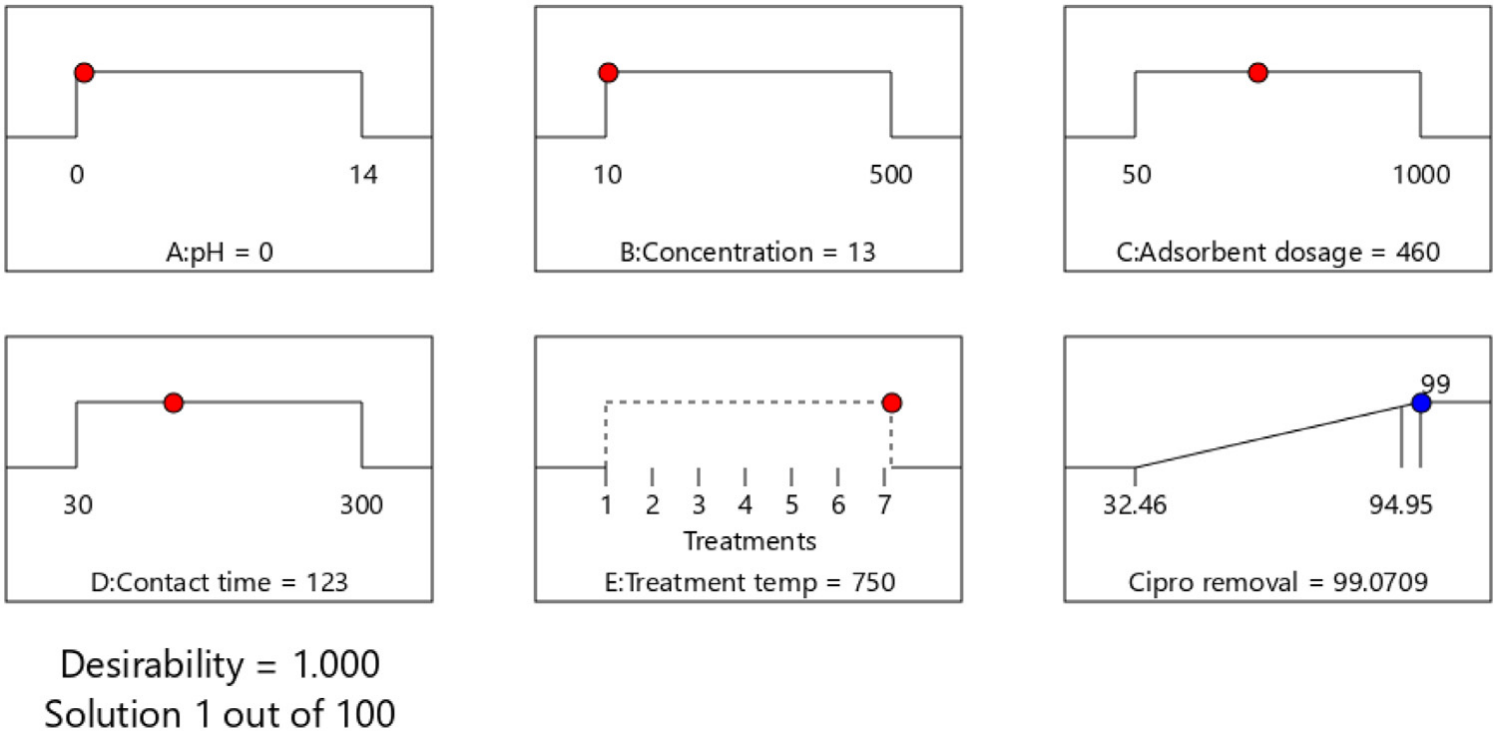
Ramps showing optimum conditions for ciprofloxacin removal efficiency.

**Fig. 17:**
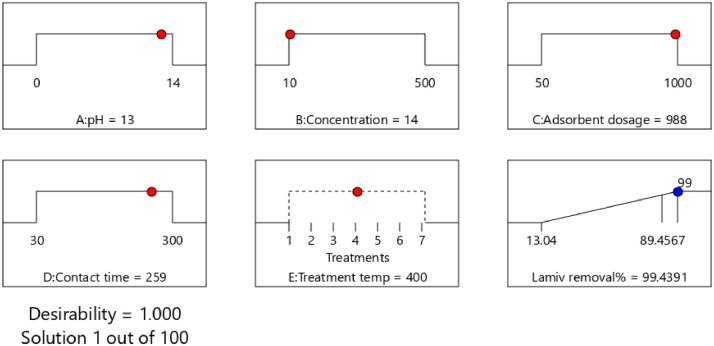
Ramps showing optimum conditions for lamivudine removal efficiency.

The adsorption conditions were numerically optimized using a desirability function of Design-Expert software to maximize removal efficiency. Using the models created during analysis, the best-operating conditions that meet the defined goals were searched within the design space. Finally, one solution among the recommended solutions was selected for the model validation, whereby three replicates of experimental runs were conducted, and the results were compared with the predicted values. Figs. 16 and 17 shows the ramps for the optimum conditions of removal efficiency of ciprofloxacin and lamivudine. The optimum removal efficiency of lamivudine (99.4%) was slightly higher compared to that of ciprofloxacin (99.1%) at different optimum conditions. Although the produced adsorbent removed almost same amount of pollutant concentrations, ciprofloxacin 13 mg/l while lamivudine 14 mg/l, it is worth noting the diversity of other factors. The adsorbent is very active in removing ciprofloxacin at 0 pH compared to 13 for lamivudine. In contrast, parameters such as adsorbent dose, contact time, and temperature were two times higher when comparing the adsorption capabilities of lamivudine and ciprofloxacin.

The validity of the predicted models was assessed by running three replicates of confirmation experiments at the selected conditions of ciprofloxacin (pH 1, concentration 17, adsorbent dosage 288, contact time 38 min, and treatment temperature 750 °C) and lamivudine (pH 14, concentration 13, adsorbent dosage 999, contact time 249 min, and treatment temperature 400 °C.). The predicted removal efficiency value at these conditions was 99.1% ciprofloxacin and 99.6% lamivudine. The Residual Standard Error (RSE) obtained using Eq. (1) was 4.4% ciprofloxacin and 9.2% for lamivudine. The RSE below 10 imply an excellent agreement of experimental values with the model predicted results. This finding indicated that the prediction error for lamivudine was slightly larger; consequently, our future research will focus more on improving the lamivudine removal efficiency model.

(1) $$\text{RSE}\;\left(\%\right)=100\;X\;\frac{\left|\left(\text{Exp}.\;\text{value}-\text{pred}.\;\text{value}\right)\right|}{\text{pred}.\;\text{value}}$$

## 3 Experimental Design, Materials and Methods

### 3.1 Design of Experiments and Statistical Analysis

Response surface methodology is an empirical modelling method for determining the interaction of multiple operating and response variables. It provides a systematic experimentation strategy for building and optimizing an empirical model. In essence, RSM is a combination of mathematical and statistical approaches suitable for modelling and analyzing problems in which the output is affected by input variables and their interactions [9], [10], [11]. Furthermore, the RSM reduces the number of experiments, costs, and time spent on physical experiments while providing adequate data for statistically acceptable conclusions [12]. In the current study, an RSM based on the optimality design was used to optimize five independent and one response variables. Independent variables studied are adsorbent dosage (50-1000 mg), calcination temperature (250, 400, 500, 600 and 750 °C), residence time (30-300 min), pH (1-14), and pollutant concentration (10-500 ppm), while the observed response was the removal efficiency (%) of ciprofloxacin and lamivudine. These variables were selected based on the data available in the literature [13], [14], [15]. D-optimality RSM comprises 55 experimental runs, out of which 45 are model points, five are replicate points, and five are lack-of-fit points. The RSM involves five steps: these are development of statistically designed experiments, followed by generating an empirical model, statistical analysis of the model, numerical optimization by using the desirability function and finally, model confirmation. The experimental run was randomized to minimize the error and effect of uncontrolled factors [16]. The observed responses were used to generate an empirical model conforming to the experimental variables. Experimental results from the 55 runs were used to determine the regression coefficient of the quadratic model using Design-Expert Version 13.0.5 software (Stat-Ease, Inc., Minneapolis, USA). The coefficient of R-squared established the accuracy of the fitted model, and the significant model terms were evaluated by the probability value (P-value) at a 95% confidence level. The contour plots were developed to show the interaction of two independent variables while holding the third variable at the central value. The geometry of the surface plots provides valuable information about the system's behaviour on the variation of the processing parameter within the design space.

All necessary equipment for the adsorption experiment, such as shakers, analytical balance, and glassware used at a research laboratory of the College of Natural and Mathematical Sciences, The University of Dodoma. Expendable materials and reagents were of analytical grade including methanol, distilled water, hydrochloric acid, sodium hydroxide, ciprofloxacin, and lamivudine standards. Jamun Seeds (*Syzygium cumini*) were collected, dried under shade, pulverized and sieved. The powder was then calcined at temperatures (250, 400, 500, 600 and 750 °C) in the presence of nitrogen gas using a carbolite tube furnace at the Nelson Mandela Institution of Science and Technology. Initial characterization of the material was conducted using flash 2000 elemental analyser for CHNS ratio, FTIR for functional group and quantacrome 1000 LSe series for porosity. A batch adsorption experiment was conducted to evaluate the removal of ciprofloxacin and lamivudine from a synthetic solution. The amount of ciprofloxacin and lamivudine that remained in the solution was evaluated using a UV-Vis instrument. The adsorption experiments, characterization, and RSM optimization were conducted according to previous studies [9,^13^,^14^,[17], [18], [19], [20].

## Ethics Statements

This work did not involve any animal or human subject in its experimentation process.

## CRediT authorship contribution statement

**Asha Ripanda:** Conceptualization, Methodology, Data curation, Visualization, Investigation, Writing – original draft. **Mwemezi J. Rwiza:** Supervision, Writing – review & editing. **Elias Charles Nyanza:** Supervision, Writing – review & editing. **Ramadhani Bakari:** Conceptualization, Methodology, Data curation, Visualization, Investigation, Writing – original draft. **Hossein Miraji:** Conceptualization, Methodology, Data curation, Visualization, Investigation, Writing – original draft. **Karoli N. Njau:** Writing – review & editing. **Said Ali Hamad Vuai:** Writing – review & editing. **Revocatus L. Machunda:** Supervision, Writing – review & editing.

## Declaration of Competing Interest

The authors declare that they have no known competing financial interests or personal relationships that could have appeared to influence the work reported in this paper.

## Data Availability

Data on Jamun Seed (Syzygium cumini) Biochar; preparation, Initial Characterization, Experimental Design and Adsorption of Ciprofloxacin and Lamivudine: Part 1 (Original data) (Mendeley Data).
